# Supplementary figures and images for: High-resolution mapping of the neutralizing and binding specificities of polyclonal sera post-HIV Env trimer vaccination
Source: eLife. 2021 Jan 13;10:e64281. doi: 10.7554/eLife.64281 (PMC7864656; doi:10.7554/eLife.64281)

| differential selection = 2

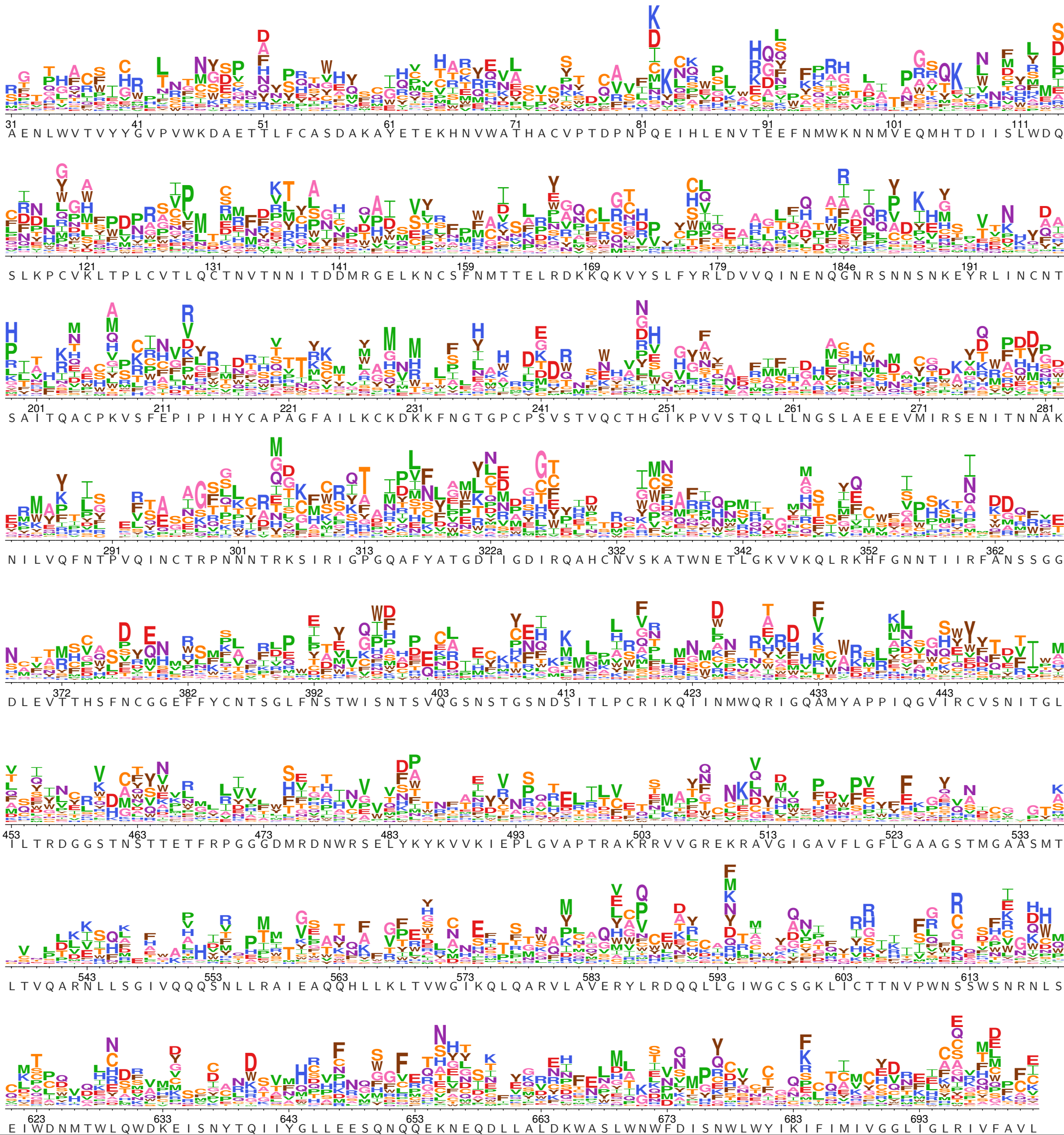

Supplement: Figure 2—source data 1. [file elife-64281-fig2-data1.zip › median-2124-Wk0_diffsel.pdf]

|differential selection = 2

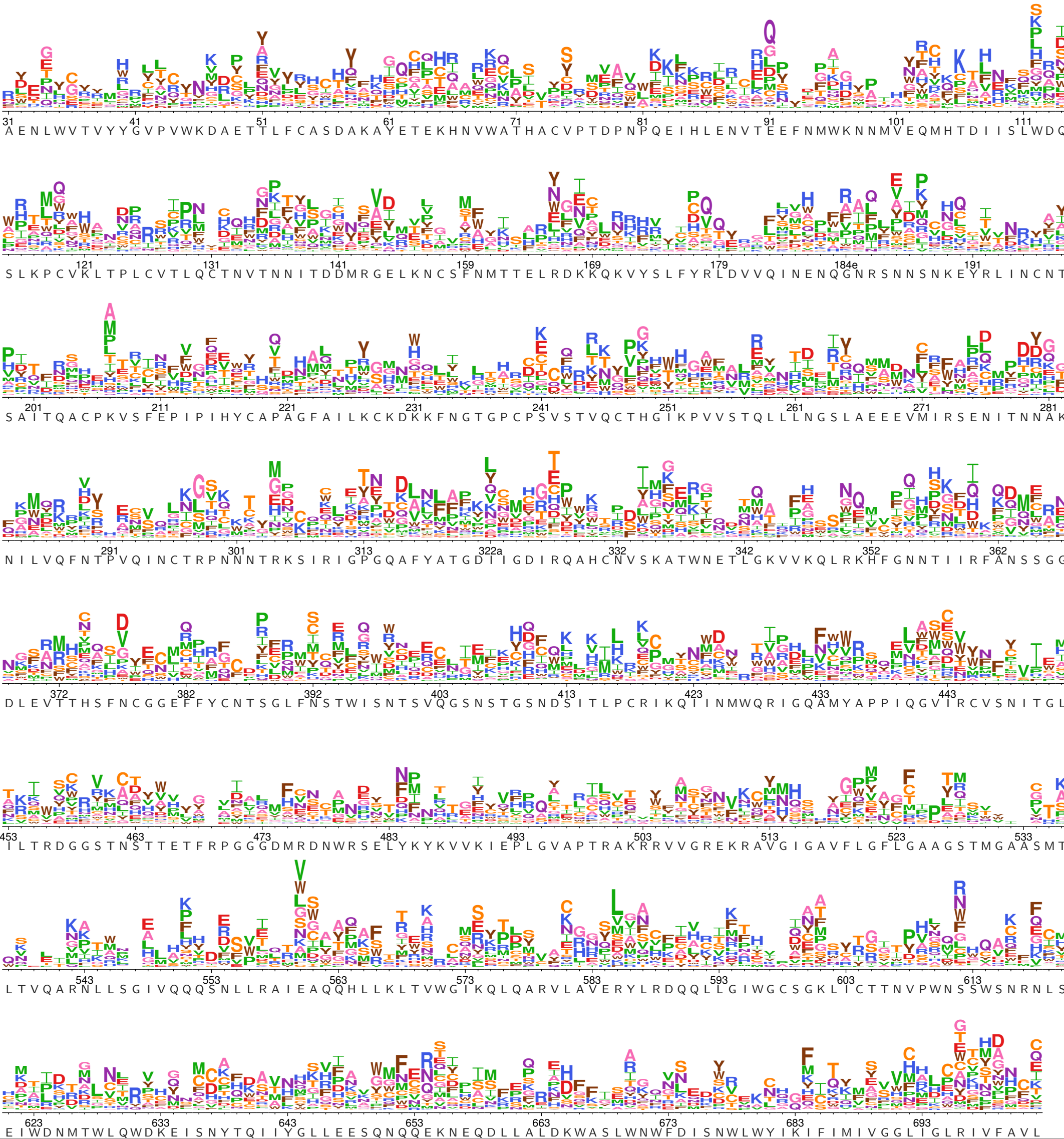

Supplement: Figure 2—source data 1. [file elife-64281-fig2-data1.zip › median-5724-Wk0_diffsel.pdf]

ldifferential selection = 4

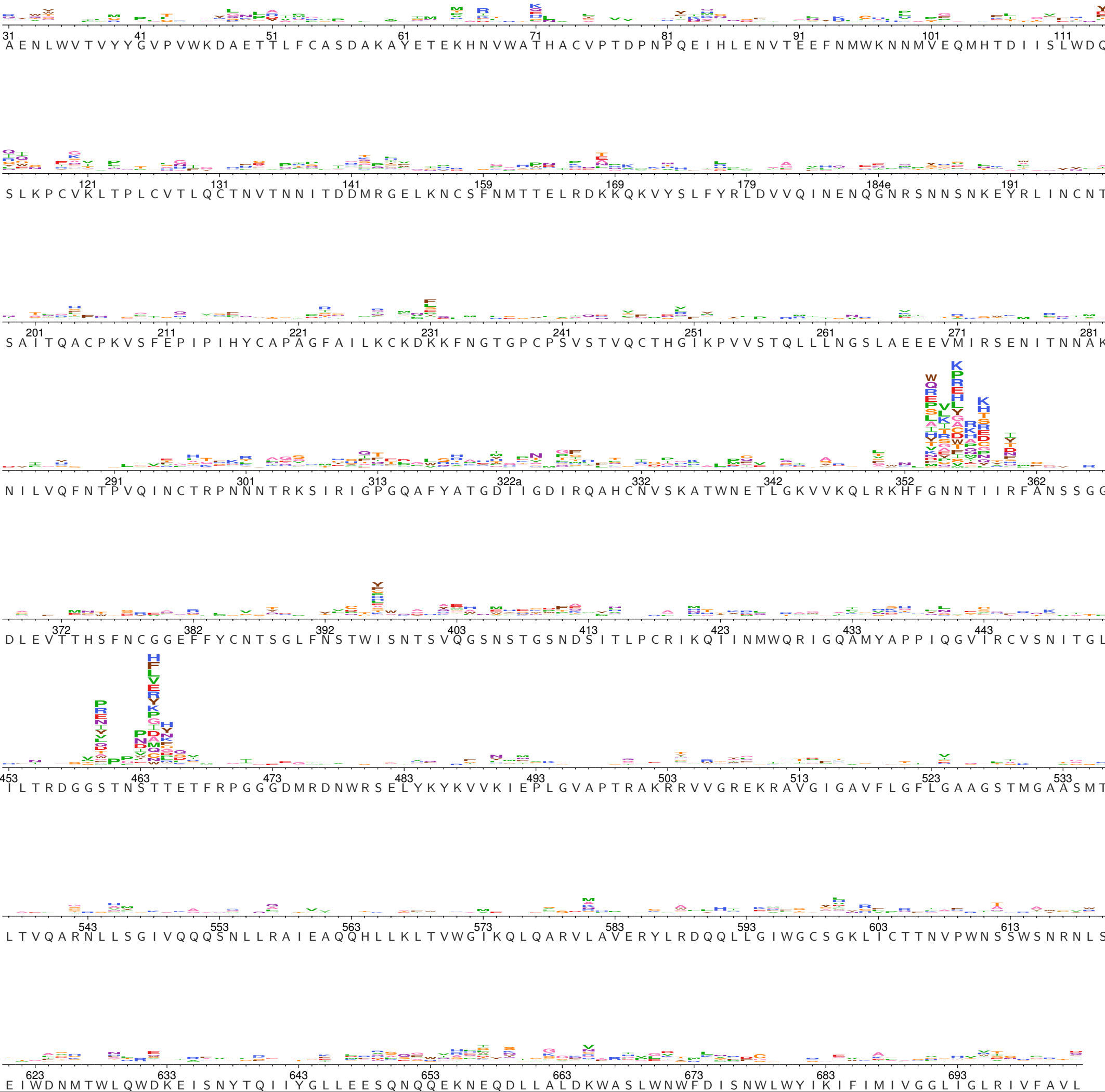

Supplement: Figure 2—source data 1. [file elife-64281-fig2-data1.zip › median-2425-Wk18_diffsel.pdf]

ldifferential selection = 1

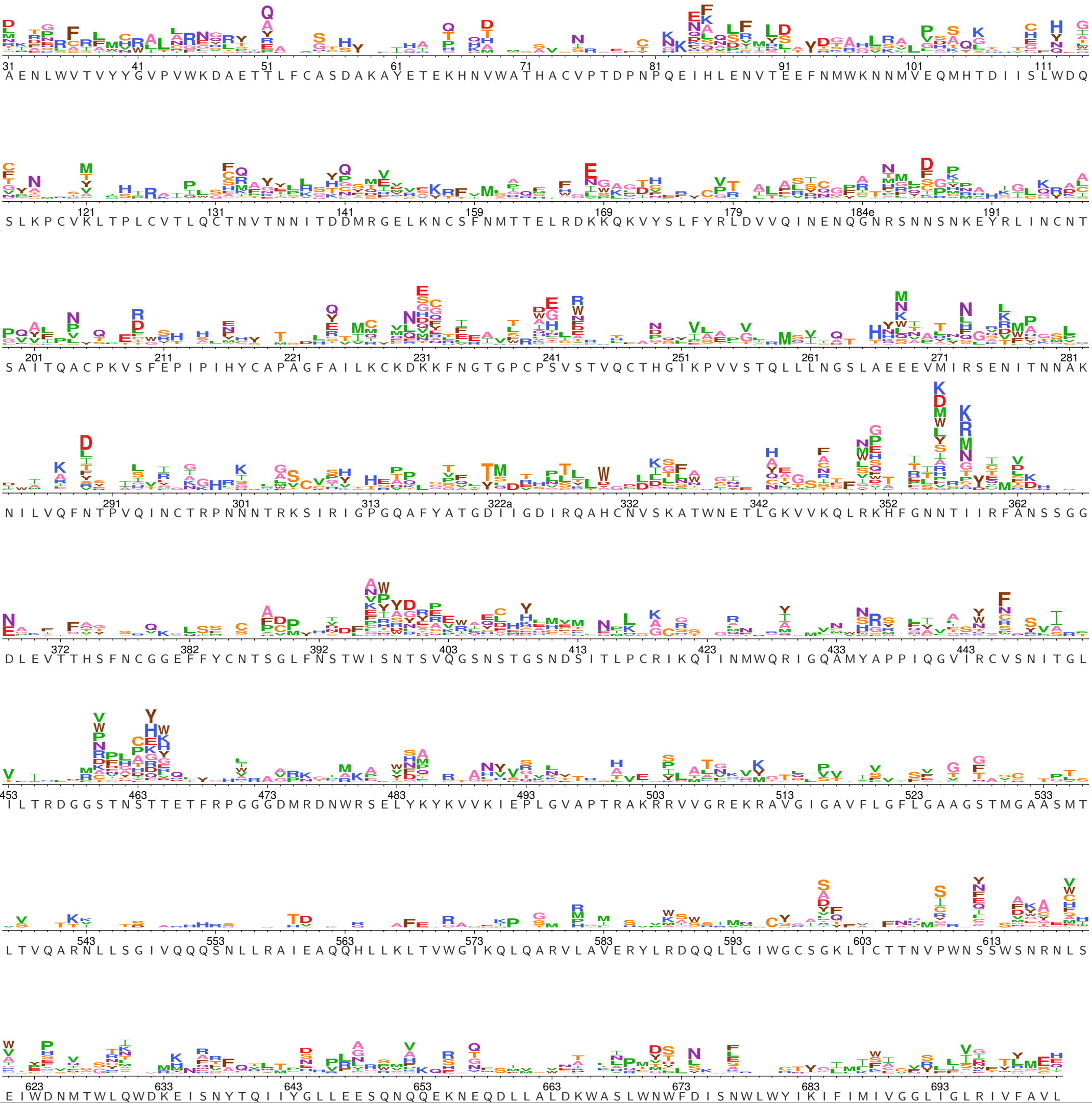

Supplement: Figure 2—source data 1. [file elife-64281-fig2-data1.zip › median-2423-Wk18_diffsel.pdf]

|differential selection = 2

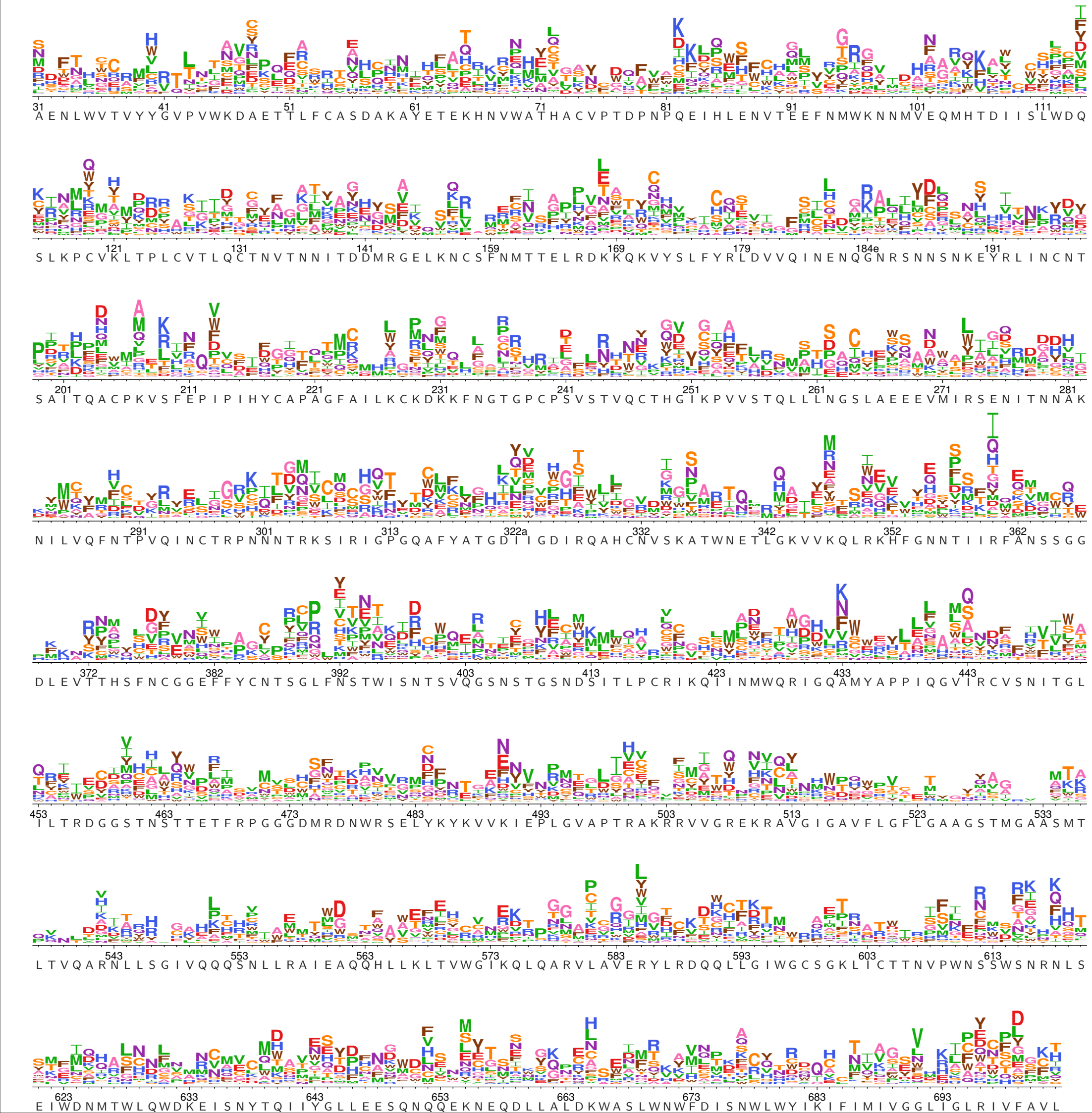

Supplement: Figure 2—source data 1. [file elife-64281-fig2-data1.zip › median-2423-Wk0_diffsel.pdf]

ldifferential selection = 2

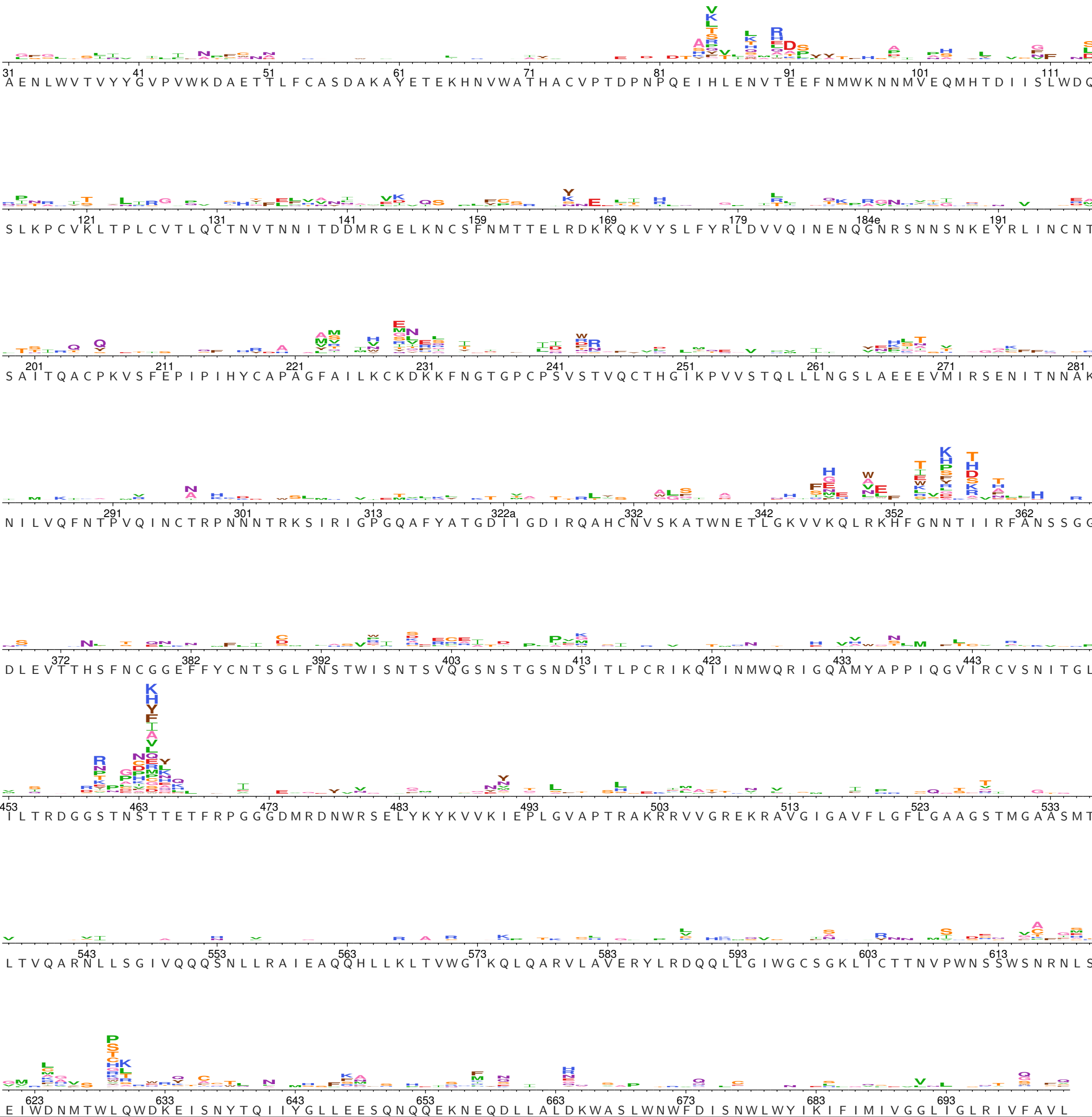

Supplement: Figure 2—source data 1. [file elife-64281-fig2-data1.zip › median-2124-Wk22_diffsel.pdf]

| differential selection = 2

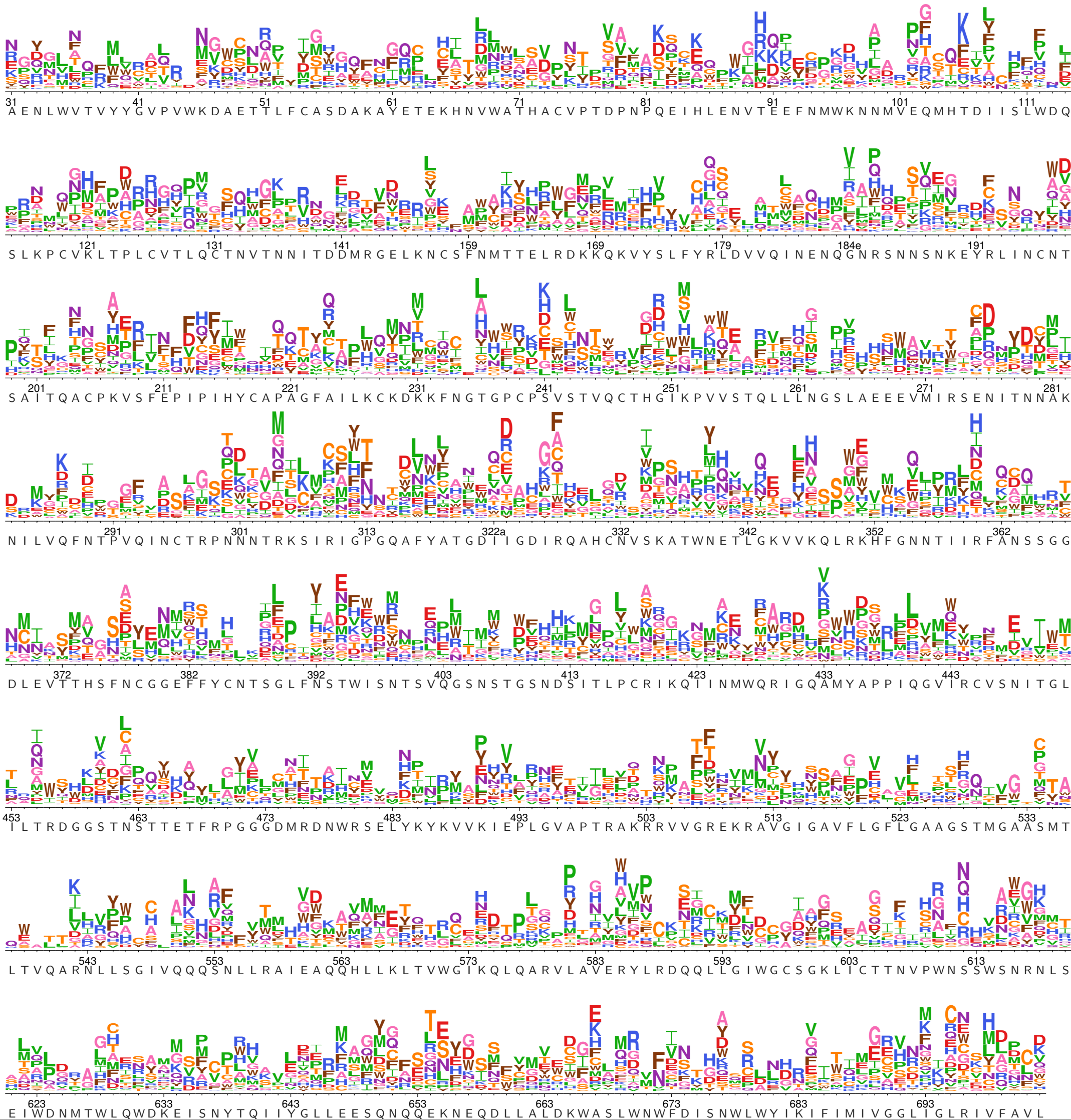

Supplement: Figure 2—source data 1. [file elife-64281-fig2-data1.zip › median-2214-Wk0_diffsel.pdf]

ldifferential selection = 3

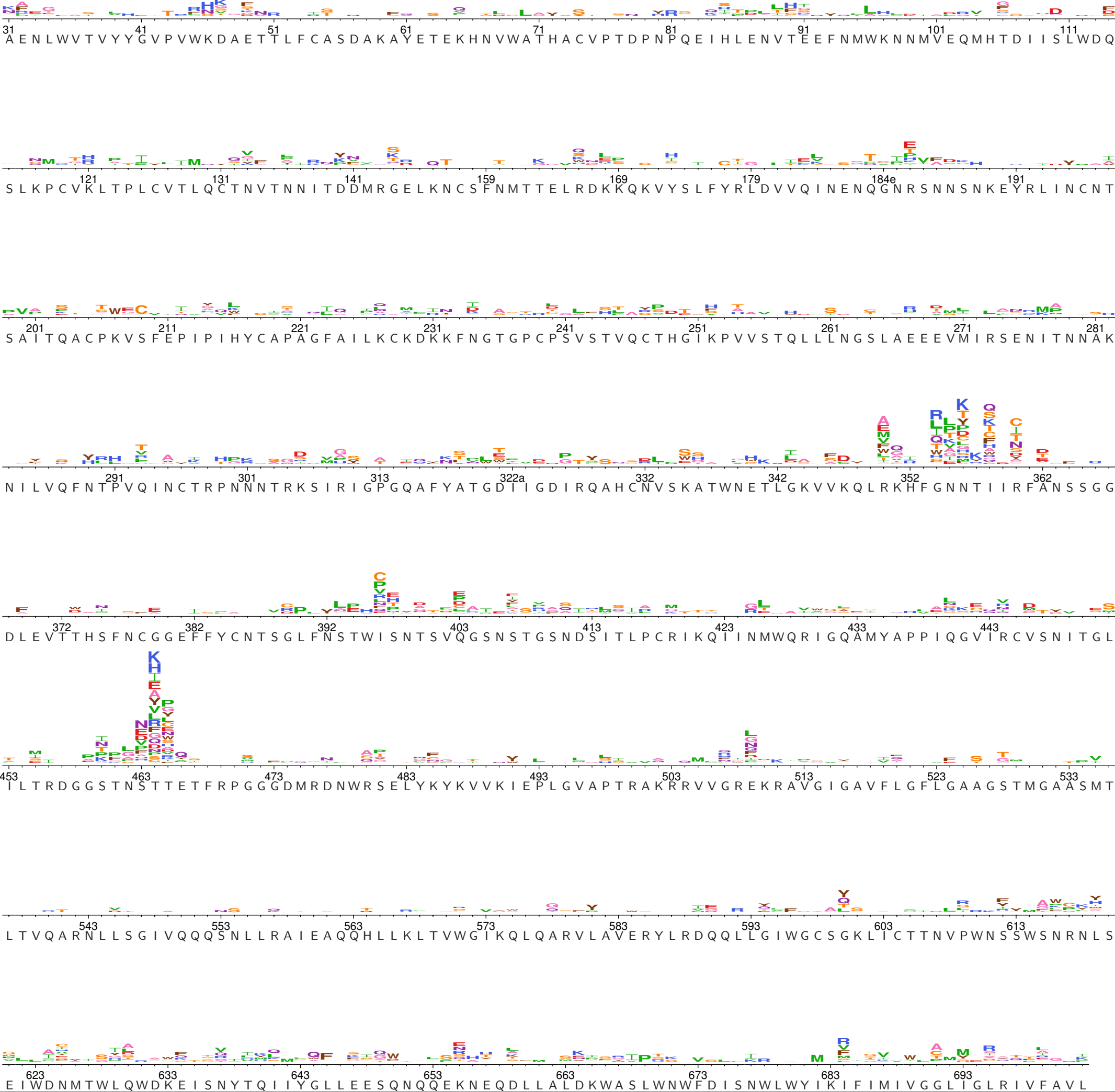

Supplement: Figure 2—source data 1. [file elife-64281-fig2-data1.zip › median-5727-Wk26_diffsel.pdf]

ldifferential selection = 8

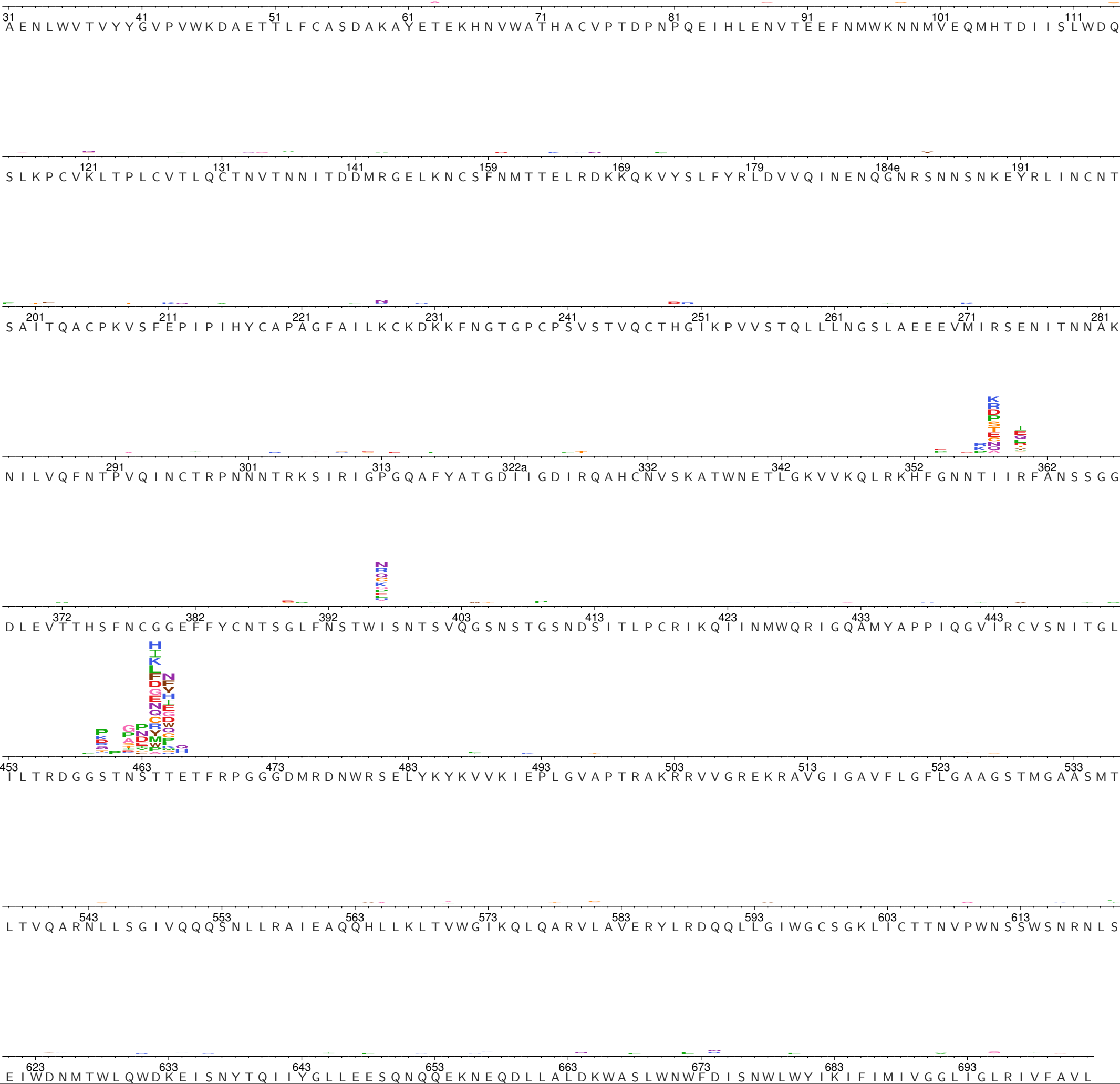

Supplement: Figure 2—source data 1. [file elife-64281-fig2-data1.zip › median-5724-Wk26_diffsel.pdf]

| differential selection = 2

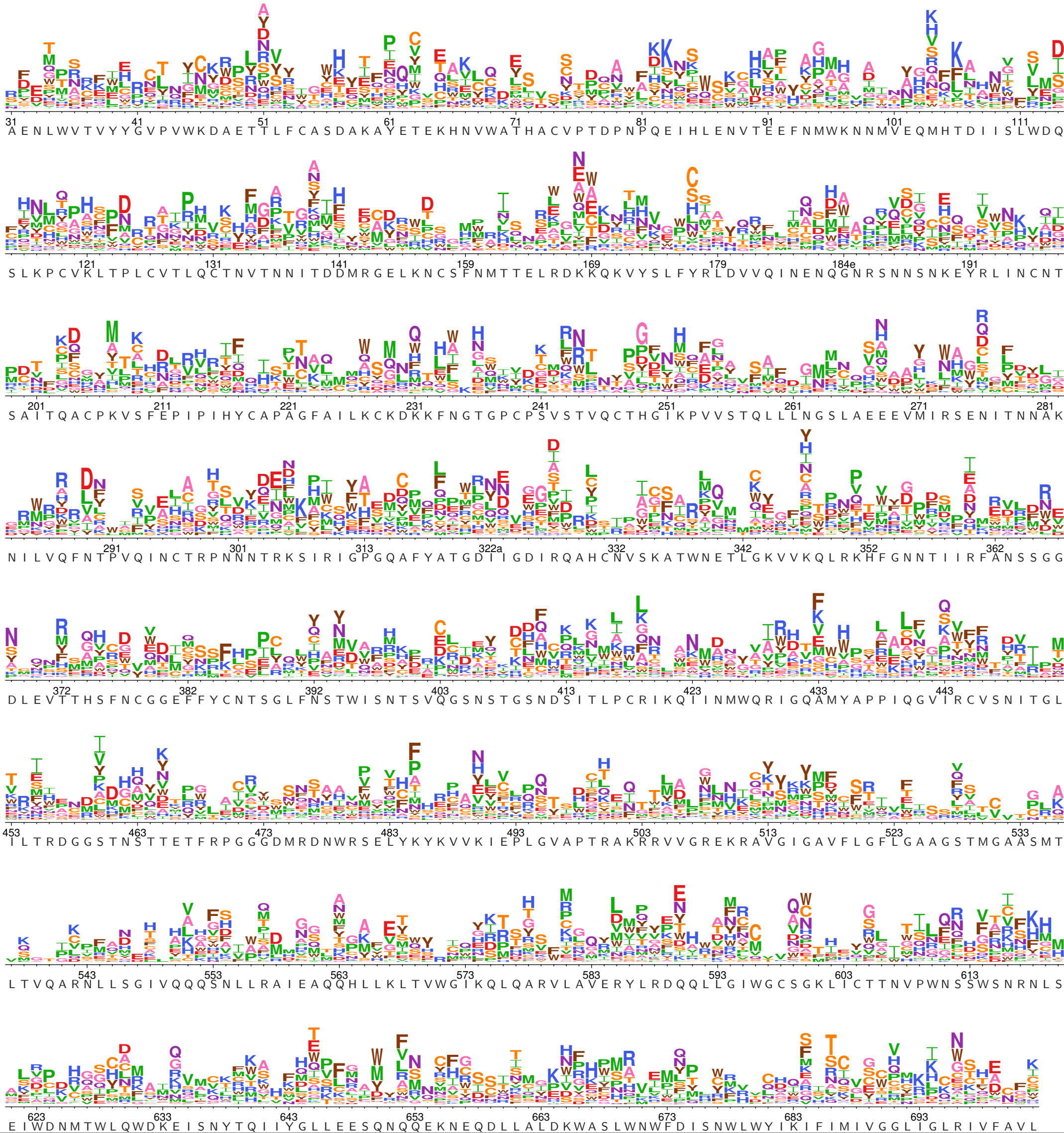

Supplement: Figure 2—source data 1. [file elife-64281-fig2-data1.zip › median-2425-Wk0_diffsel.pdf]

| differential selection = 2

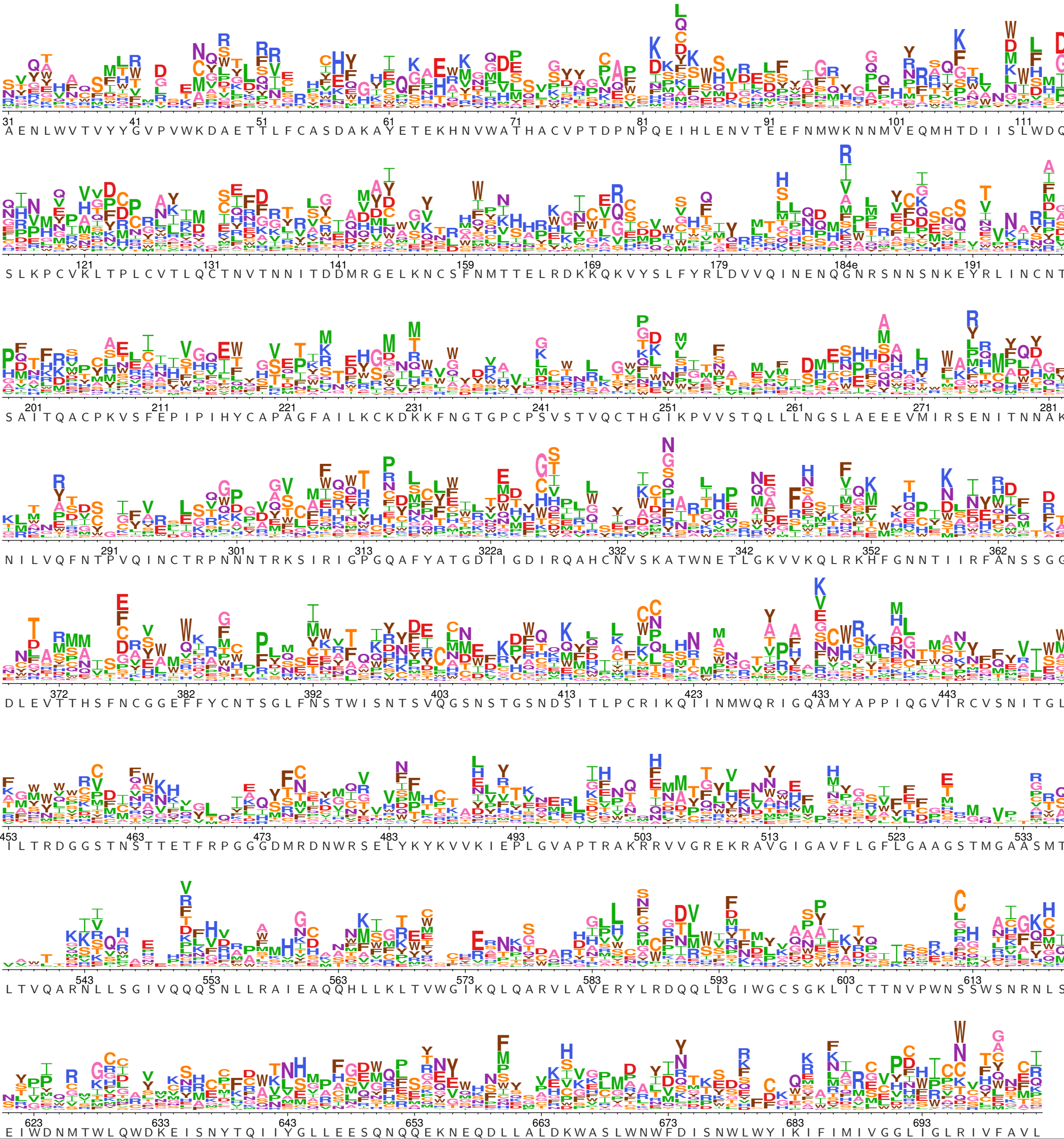

Supplement: Figure 2—source data 1. [file elife-64281-fig2-data1.zip › median-5727-Wk0_diffsel.pdf]

|differential selection = 1

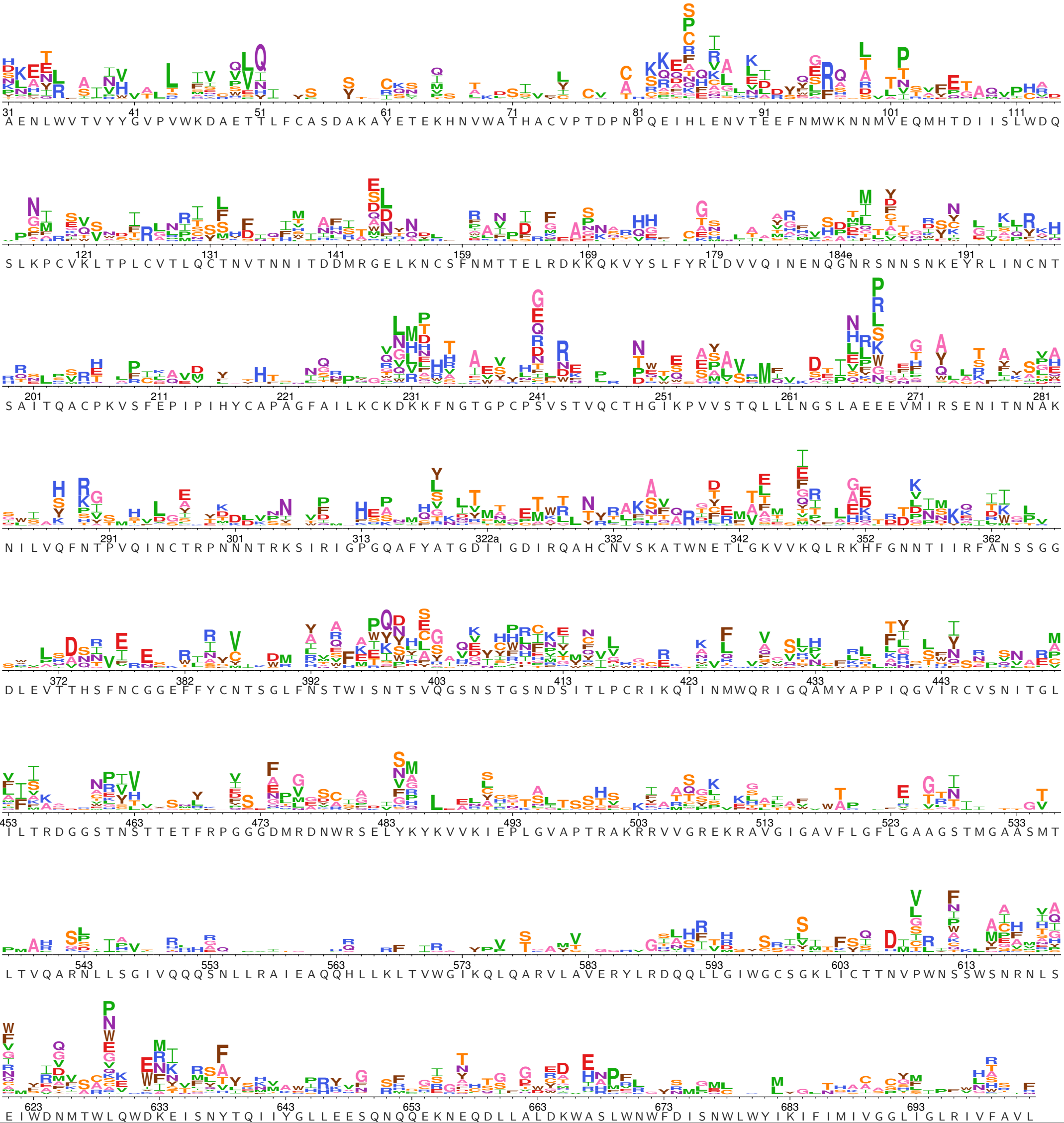

Supplement: Figure 2—source data 1. [file elife-64281-fig2-data1.zip › median-2214-Wk43_diffsel.pdf]
